# Simplified Heart Failure Clinic Questionnaire: A Practical Patient-Reported Symptom Survey

**DOI:** 10.7759/cureus.113524

**Published:** 2026-07-28

**Authors:** Karleena Snow, Arindam Sharma

**Affiliations:** 1 Cardiology, Heart Failure, University of Utah, Salt Lake City, USA; 2 Advanced Heart Failure, Intermountain Health, St. George, USA

**Keywords:** free heart failure questionnaire, heart failure case report, heart failure management tool, patient-reported outcomes measurement, simplified heart failure questionnaire

## Abstract

Managing chronic heart failure (HF) is challenging for clinicians across a variety of specialties. Many of the currently available symptom questionnaires are complex, time-consuming for patients to complete, and costly for providers or institutions to use. We created a simplified questionnaire that patients could easily use in a clinic setting, that would be free for the clinician/institution to use, and that would improve symptom assessment and better guide HF treatment. We implemented it in a pilot program.

During our pilot study, we used the Simplified Heart Failure Clinic Questionnaire (SHFCQ) in over 1,000 patients. Still, we give three case examples in this article to show the benefits of using the symptom questionnaire within a variety of HF types. Each patient was given the SHFCQ during their HF clinic visits, and they were educated on its use. Clinicians and patients were able to give feedback about the questionnaire throughout the program, and the SHFCQ underwent three revisions to improve functionality and usability.

This illustrative article highlights the preliminary use of the SHFCQ. We found that patients were very receptive to it and that it improved the providers’ ability to dive deeper into the patient’s reported symptoms and create a more tailored treatment plan. We feel that it has the potential to become a free, easy-to-use tool in the management of HF clinic patients. Further large-scale study is needed to validate the questionnaire’s reliability and reproducibility.

## Introduction

Treating chronic heart failure (HF) poses a challenging task for all types of clinicians due to the varied HF etiologies, responses to guideline-directed medical therapy (GDMT), the wide range of presenting symptoms, and the limited time available during clinic appointments. Various symptom surveys have been created to help determine how the patient is responding to treatment and the symptom burden on their quality of life. We have found that many of these questionnaires are complex, time-consuming for patients to complete, and costly for providers or institutions to use. We created a simplified patient-reported symptom questionnaire that patients in a clinic setting could easily use, would be free for clinicians and institutions to use, and would help improve symptom assessment to better guide HF treatment. We implemented this new questionnaire as a pilot program in our HF clinics to gauge its usefulness for patients reporting their symptoms and for providers assessing symptom burden.

As HF providers, we are aware that HF is a complex syndrome with high morbidity and mortality and is currently estimated to affect more than 64 million people worldwide [1], with an estimated 6.5 million HF patients in the United States [2]. There are only around 1,000 board-certified advanced HF and transplant cardiology specialists in the United States, and a large burden of caring for these complex patients falls on general cardiologists and primary care providers [2]. Using a patient-reported symptom burden questionnaire can help providers in a variety of clinics assess and treat their HF patients.

We created the Simplified Heart Failure Clinic Questionnaire (SHFCQ) in 2024. We introduced it as a pilot program in two advanced HF clinics in Utah, after finding that many of the other available questionnaires were expensive to use or burdensome to the patient and/or clinician. It was created using the most common symptoms our HF patients reported being bothered by, symptoms we commonly asked about at each clinic visit. During the two-year pilot program, we utilized the questionnaire daily in our HF clinics, with over 1,000 unique patients. We aimed to use this pilot program to help fine-tune the questionnaire to make it concise and easy to use for both the patient and the provider. While it has not yet been formally validated, our unique symptom questionnaire underwent three revisions after obtaining feedback from physicians, nurse practitioners, registered nurses, medical assistants, and clinic patients. We used the questionnaire with all of our HF clinic patients. Still, we present three illustrative patient anecdotes in which the SHFCQ demonstrated its usefulness in treating HF with reduced ejection fraction (HFrEF), HF with preserved ejection fraction (HFpEF), and biventricular (BiV) failure.

## Case presentation

**Figure 1:**
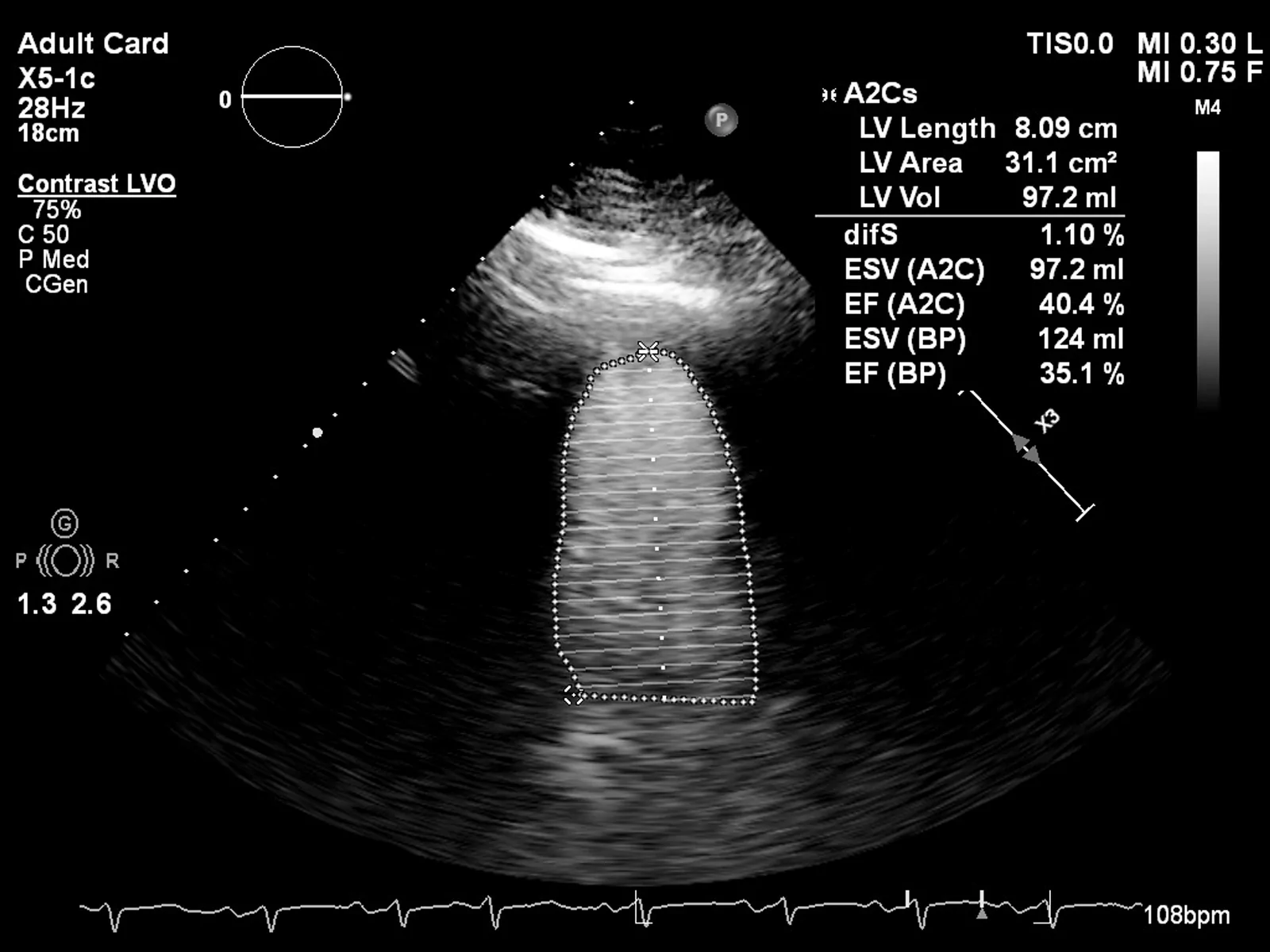
Transthoracic echocardiogram for Patient A showing reduced ejection fraction

**Figure 2:**
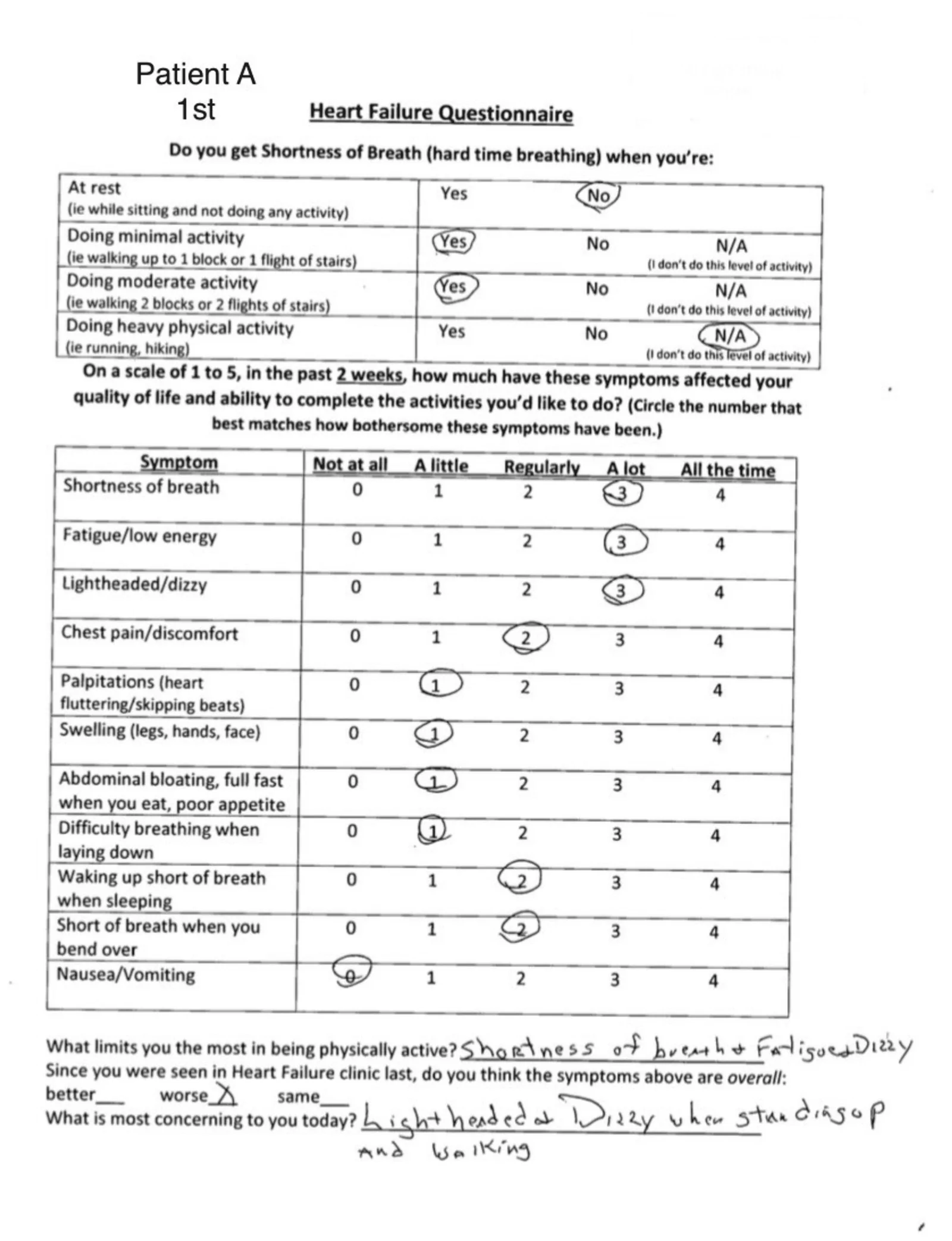
Patient A's first SHFCQ SHFCQ: Simplified Heart Failure Clinic Questionnaire

**Figure 3:**
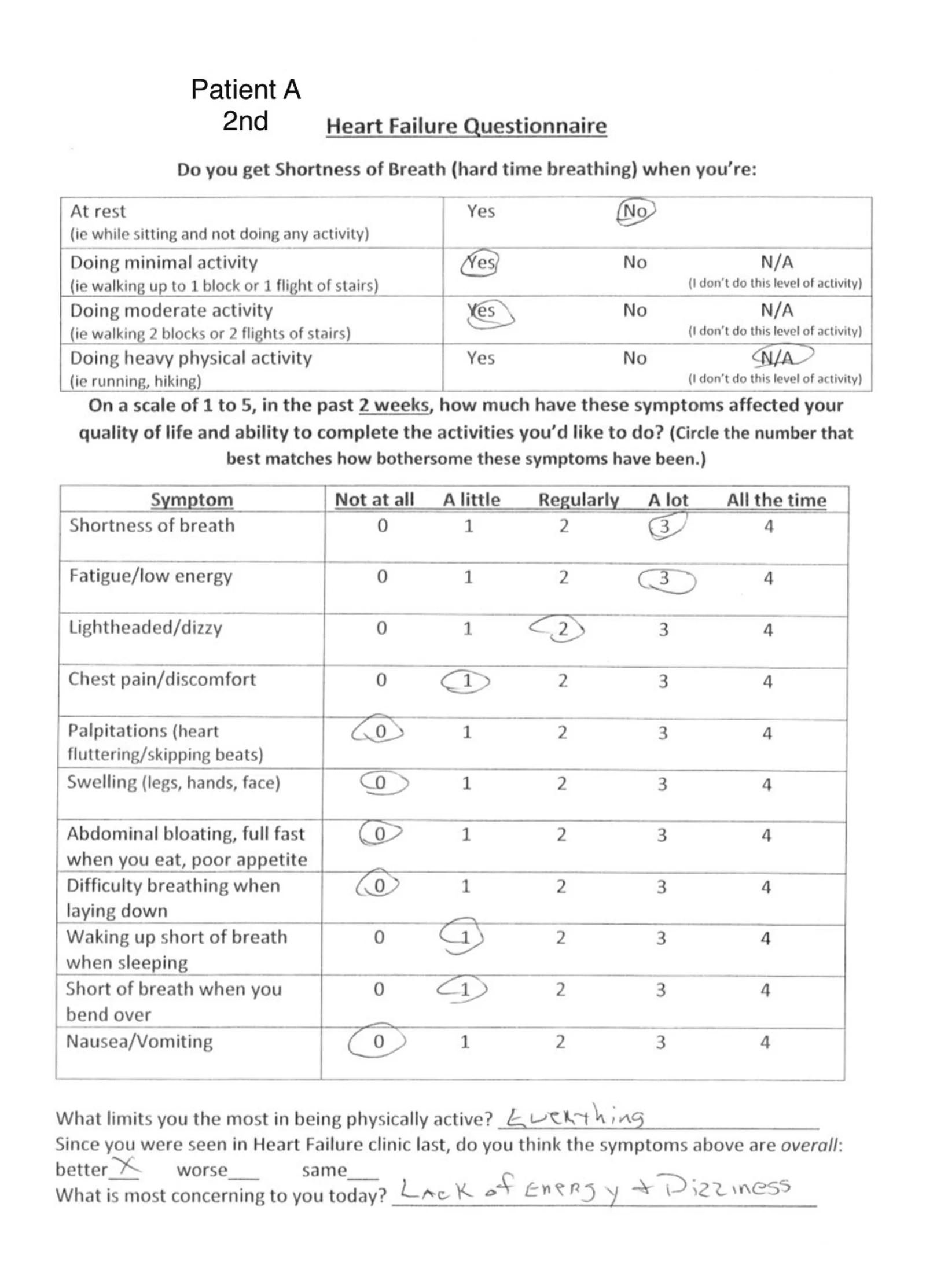
Patient A's second SHFCQ after interventions SHFCQ: Simplified Heart Failure Clinic Questionnaire

Patient A (HFrEF) is a 67-year-old man who developed HFrEF in the setting of nonischemic cardiomyopathy due to tachycardia from rapid atrial fibrillation. His ejection fraction had declined to 35% (Figure 1). He came to the HF clinic after a recent hospitalization and reported feeling poorly, with his biggest complaints being increased lightheadedness, fatigue, and persistent dyspnea (Figure 2). His answers on the SHFCQ prompted a more focused survey and exam in the clinic, and he was found to be hypovolemic after aggressive diuresis in the hospital. His oral diuretic was changed to flexible as-needed dosing, and he was encouraged to increase his fluid intake. He returned to the HF clinic two weeks later and noted a gradual improvement in his functional status compared to prior (Figure 3). Subsequent appointments, with completed questionnaires, showed continued lightheadedness and prompted further evaluation with additional questioning of non-cardiac causes of lightheadedness. This triggered him to remember a prior traumatic injury affecting his ears, and a referral to an ear, nose, and throat specialist was ordered.

**Figure 4:**
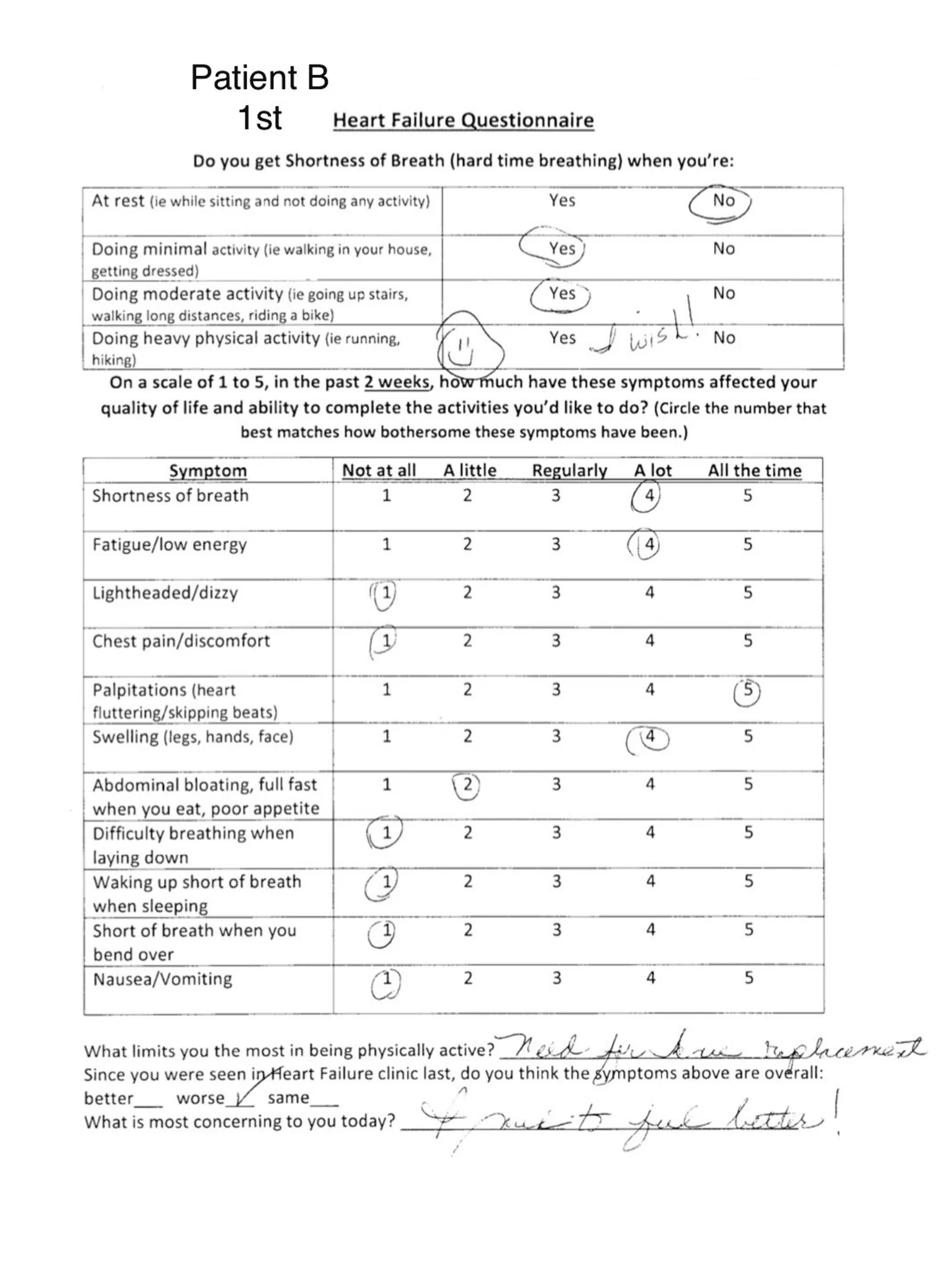
Patient B's first SHFCQ using an older version of the SHFCQ SHFCQ: Simplified Heart Failure Clinic Questionnaire

**Figure 5:**
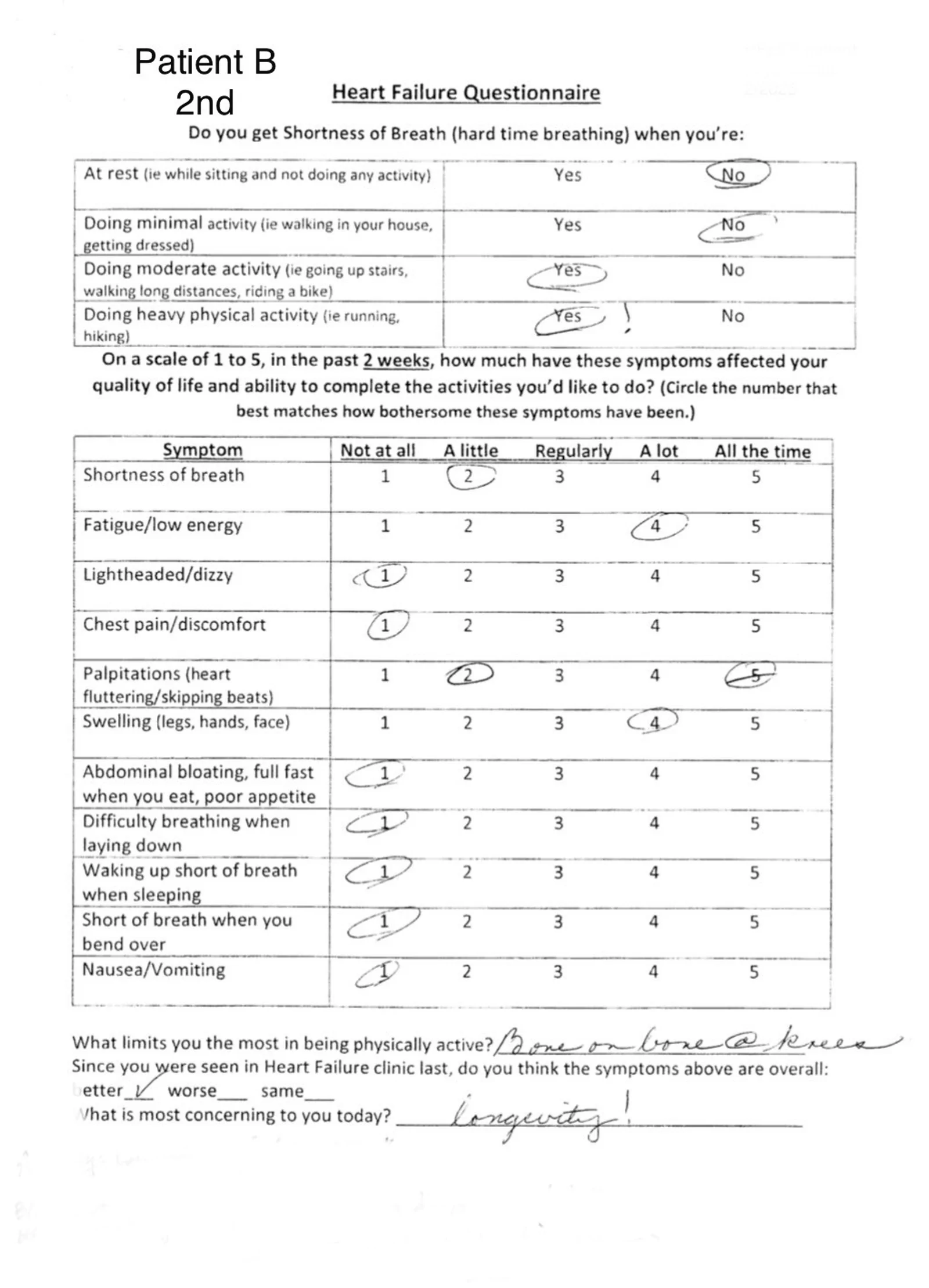
Patient B's second SHFCQ after interventions using an older version of the SHFCQ SHFCQ: Simplified Heart Failure Clinic Questionnaire

Patient B (HFpEF) is a 77-year-old woman who had been followed for several years by the HF clinic and had a complicated history and symptoms that were difficult to manage. She came to the HF clinic reporting bothersome dyspnea, fatigue, edema, and bloating despite being intravascularly euvolemic, hemodynamically stable, and on optimal GDMT. Her severe symptom answers on the questionnaire suggested that something significant was going on despite her previously stable HFpEF (Figure 4). It prompted further evaluation, and she was found to have worsening renal dysfunction (Table 1) and worsening pulmonary arterial hypertension. Appropriate medication changes were made, and she was referred to nephrology for further workup. She was also able to follow up with the pulmonary arterial hypertension clinic and started therapy with macitentan. When she was seen again six months later, her SHFCQ showed significant improvement in her dyspnea and fatigue, and she reported overall clinical improvement (Figure 5). Utilizing the questionnaire helped HF providers to investigate her ongoing symptoms and refer her to other teams to help manage her complicated comorbidities.

**Table 1:** Patient B's worsening renal function lab values, with normal ranges shown, her prior baseline, and her labs at the time of the visit when she was feeling poorly

Renal function	Normal ranges	Patient’s prior baseline	Patient’s worsening labs at time of visit
Sodium	136-145 mmol/L	139	140
Potassium	3.5-5.1 mmol/L	4.2	4.8
Chloride	96-111 mmol/L	103	108
CO2	20-30 mmol/L	27	23
Anion gap (Na+K)-(Cl+CO2)	6-18 mmol/L	9	9
Glucose	70-99 mg/dL	86	97
Blood urea nitrogen	6-24 mg/dL	51	56
Creatinine	0.53-1.26 mg/dL	1.74	2.62
Glomerular filtration rate	>60 mL/min/1.73m2	30	18
Calcium	8.3-10.1 mg/dL	9.4	9.9

**Figure 6:**
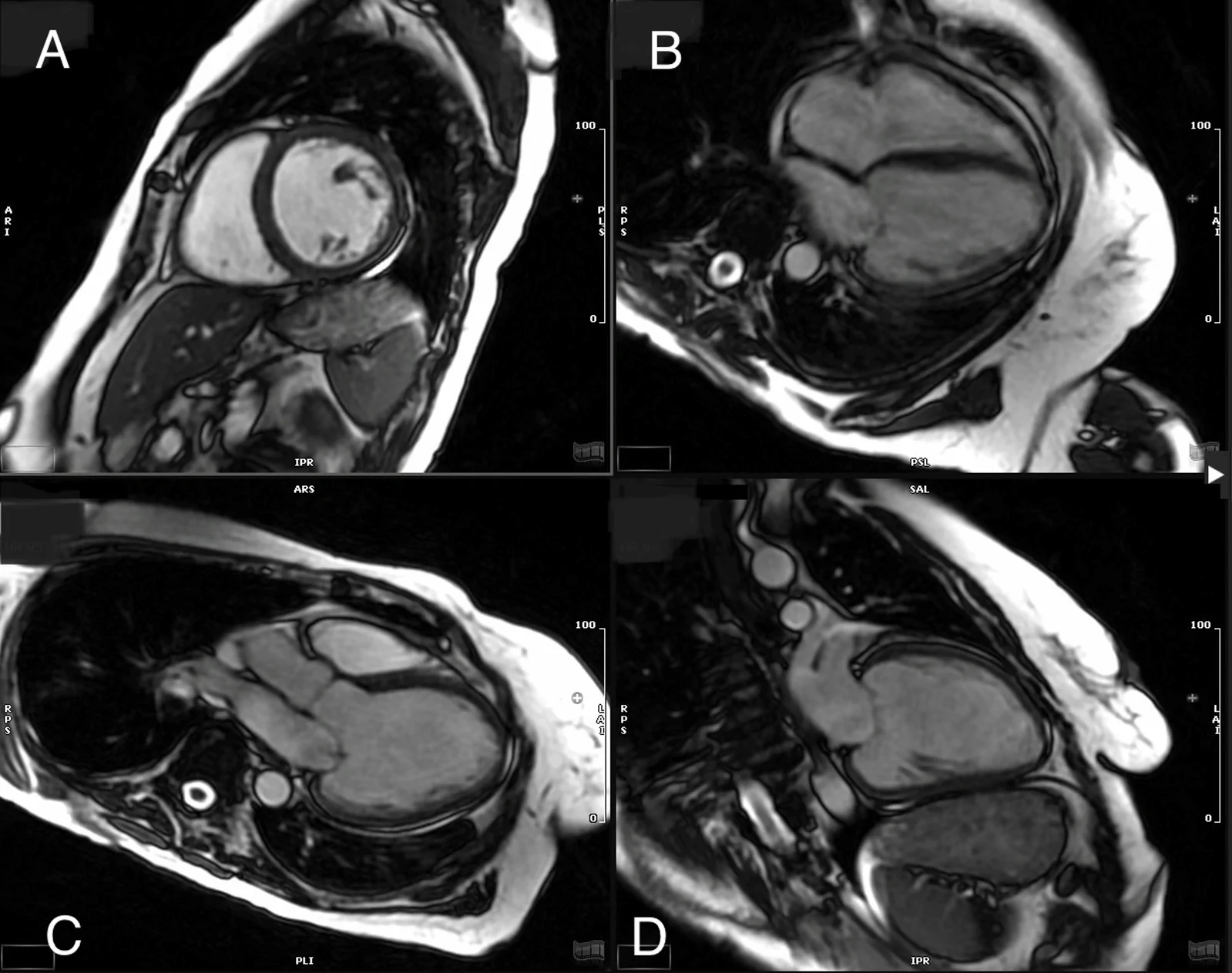
MRI of Patient C's BiV failure (A) Short-axis view. (B) Four-chamber view. (C) Three-chamber view. (D) Two-chamber view. MRI: magnetic resonance imaging, BiV: biventricular

**Figure 7:**
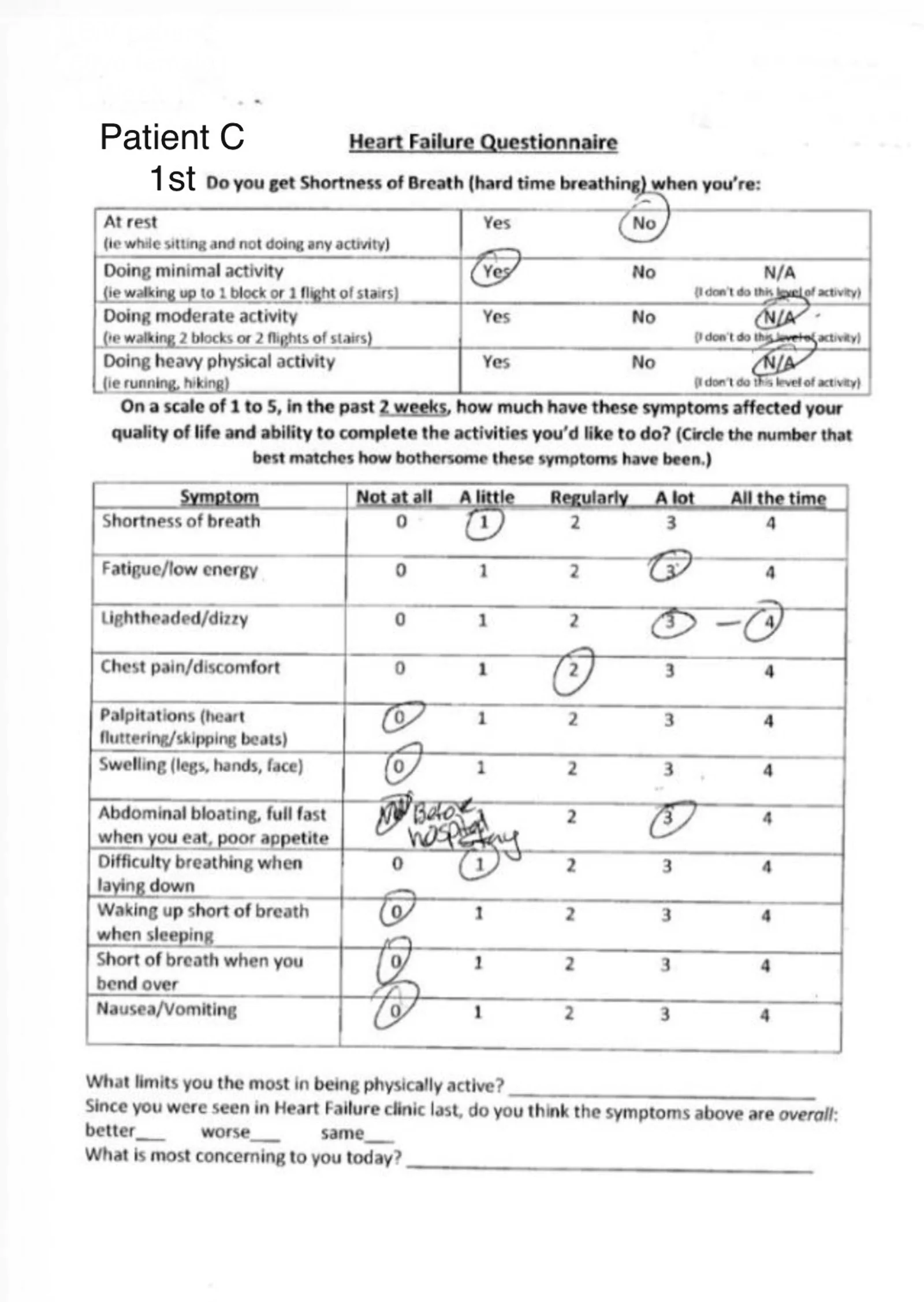
Patient C's first SHFCQ SHFCQ: Simplified Heart Failure Clinic Questionnaire

**Figure 8:**
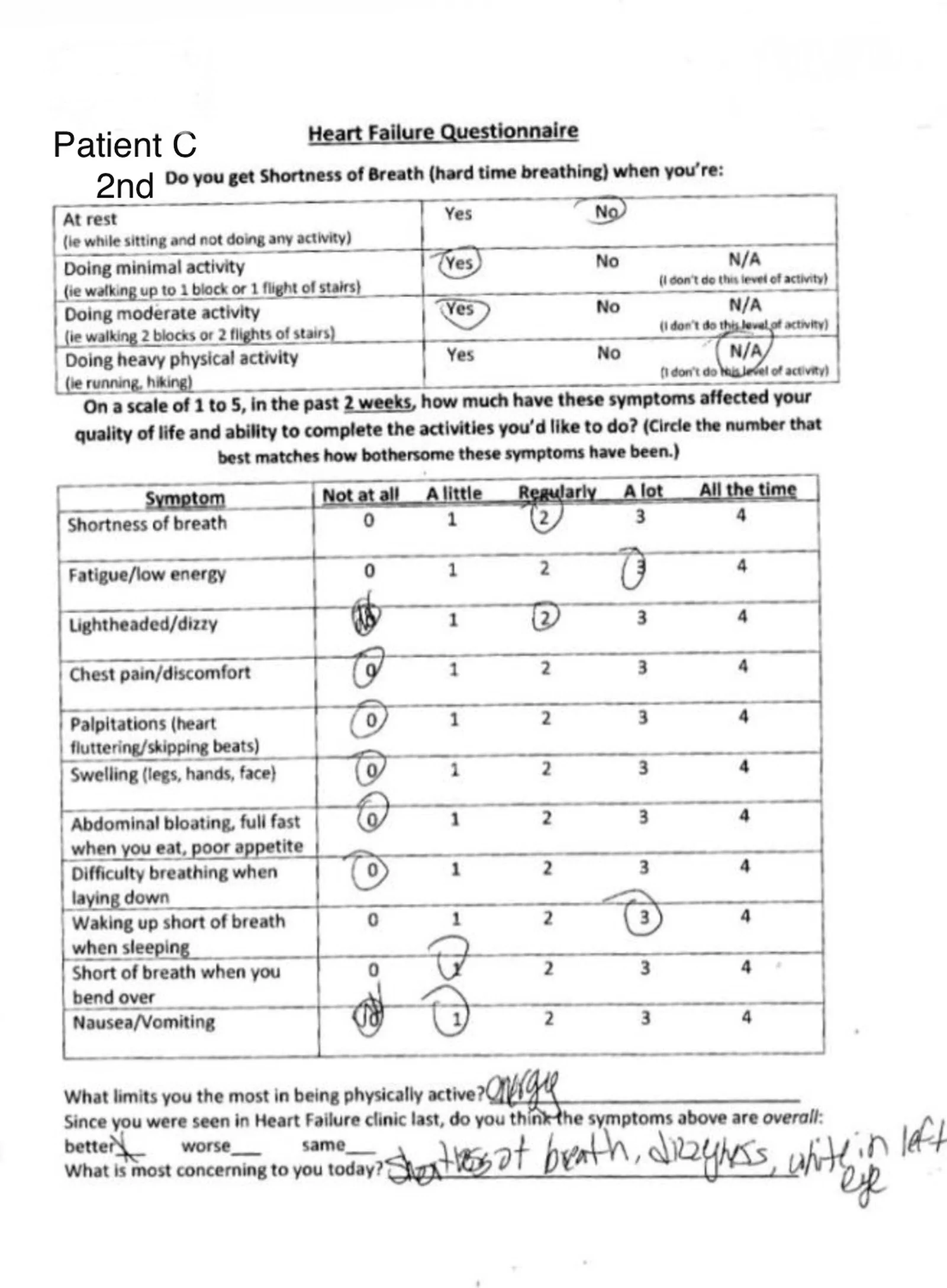
Patient C's second SHFCQ after interventions

Patient C (BiV failure) is a 68-year-old woman who was hospitalized after developing worsening dyspnea, chest pressure, edema, and weight gain and was found to have BiV failure and acute decompensation. She underwent an extensive workup, and her cardiac MRI showed left ventricular EF of 19% and right ventricular EF of 16% (Figure 6). She was only able to start on minimal GDMT due to hypotension. When she presented to the clinic one week post-hospitalization, she verbally told the provider she was feeling “a lot better.” Her symptom questionnaire showed otherwise, however, noting that she was still quite fatigued and had dyspnea with minimal exertion, lightheadedness, random chest pains, and brain fog (Figure 7). Had we only gone off her saying she was feeling “better,” we may not have dug deeper in the appointment to discover that she still needed more optimization, as evidenced by her questionnaire answers. She was found to be hypovolemic and still mildly hypotensive, and lab work showed low iron levels (Table 2). Her diuretic was changed to as-needed dosing, and iron infusions were ordered. At her follow-up clinic appointments, her SHFCQ showed improvement in her prior symptoms, and she was working to increase her physical activity (Figure 8).

**Table 2:** Iron studies for Patient C TIBC: total iron-binding capacity

Iron panel	Normal ranges	Patient values
Iron	37-170 mcg/dL	16 mcg/dL
TIBC	238-453 mcg/dL	449 mcg/dL
Iron saturation	20-50%	4%
Ferritin level	10-200 ng/mL	21 ng/mL

Our HF patients often come to the clinic overwhelmed with a variety of symptoms and concerns, and it’s easy for them to forget to address all of them. This questionnaire prompted them to remember changes they’d been feeling and most bothersome symptoms. They felt confident that the provider would see and address these concerns because the paper questionnaire was handed directly to the providers and discussed during the visit.

Table 3 shows a comparison of all three patients’ overall scores, the changes in their New York Heart Association (NYHA) score, and how they felt their symptoms were compared to the prior visit. By using the SHFCQ at every visit, subsequent questionnaires can be visually compared to the prior questionnaires to see if progress is being made. Scores can be tallied to see if the overall total is improving (Table 3), or providers can even just take a quick look at the pattern of numbers circled (more circles on the right suggest a high burden of symptoms; more circles on the left suggest a low burden, as shown with the actual patient-completed questionnaires above). The questionnaires prompted providers to focus on the higher circled numbers, see patterns over time, and note when something seems different from expected.

**Table 3:** Comparison of the three patient's SHFCQ scores in their first visit compared to the second after interventions HFrEF: heart failure with reduced ejection fraction, HFpEF: heart failure with preserved ejection fraction, BiV: biventricular, SHFCQ: Simplified Heart Failure Clinic Questionnaire, NYHA: New York Heart Association

	First SHFCQ totals	First NYHA score	Second SHFCQ totals	Second NYHA score	Overall reports feeling better, worse, or the same
Patient A (HFrEF)	19	III	11	III	Better
Patient B (HFpEF)	25	III	19	II	Better
Patient C (BiV failure)	14	III	12	III	Better

## Discussion

These cases suggest that the SHFCQ could be a useful assessment tool in the care of patients with HF. Clinic providers were better able to help their patients improve their symptom burden because they had this form completed by the patient at each appointment. It also helped providers identify additional conditions affecting symptoms that might otherwise have been attributed to HF and prompted appropriate referrals to other specialties.

According to Savarese et al. [3], patient-reported outcomes have a good association with prognosis and evaluation of disease status in multiple randomized controlled trials. Still, they have not been readily implemented into many clinics. We believe this is partially due to the difficulty of accessing the variety of questionnaires currently available. The SHFCQ was created specifically for use in clinics managing chronic HF patients. It focuses on some of the more common symptoms that affect the NYHA classification, which is commonly used to determine staging and prognosis in HF [4]. It differs from other questionnaires because it is a one-page form that is easy for the patient to complete by circling numbers. It is simple and fast for the clinician to evaluate the symptom burden based on the pattern of circles the patient made. Once it has been fully validated, it can be offered free of charge for clinicians and institutions to use.

**Figure 9:**
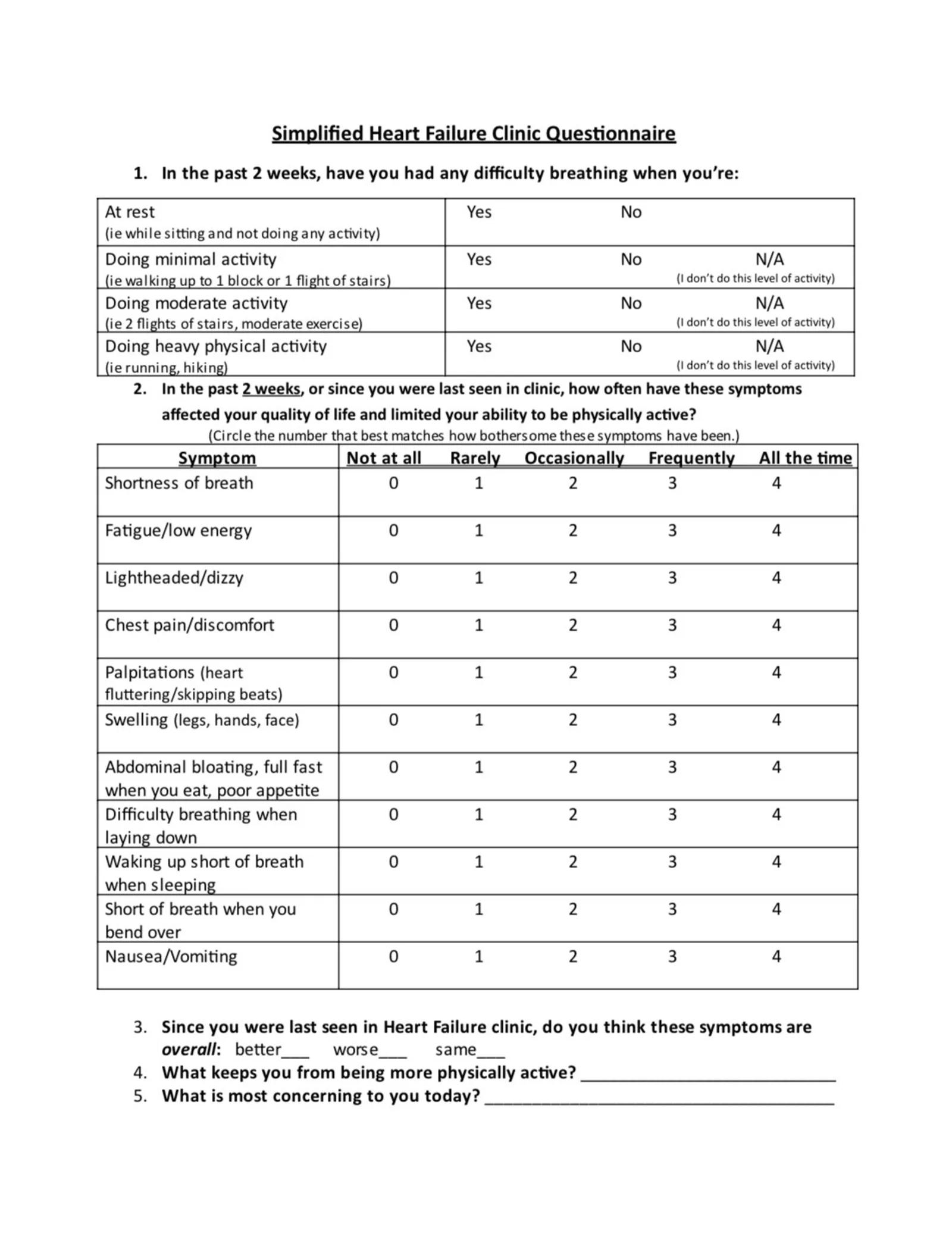
SHFCQ Created by Karleena Snow (2024). Used with permission. SHFCQ: Simplified Heart Failure Clinic Questionnaire

The first section of the SHFCQ asks about dyspnea at rest and at various activity levels, as dyspnea is one of the more classic symptoms that affect HF patients and can help with determining the NYHA classification. The next symptom section focuses on how often a variety of symptoms have affected the patient’s quality of life on average over the past two weeks, using a Likert scale from 0 (not at all) to 4 (all the time). It addresses shortness of breath, fatigue, lightheadedness, chest pain, palpitations, edema, bloating/early satiety, orthopnea, paroxysmal nocturnal dyspnea, bendopnea, and nausea/vomiting. The last section asks three questions with space for patients to write their own answers if they wish. These questions address what limits them most from being more physically active, as it isn’t always HF symptoms (i.e., it could be joint/pain, another disability, or motivation-related). It also asks what they are most concerned about at that time and how their symptoms currently compare to when they were last seen in the clinic. See Figure 9 for the full questionnaire.

The SHFCQ can help clinicians better guide the questions asked and physical exam performed in the clinic, with a focus on new symptoms, worsening ones, or those most distressing to the patient. This allows for a more targeted assessment to help tease out what could be causing the symptoms and guide the treatment. Our providers were able to evaluate symptom burden more consistently and more thoroughly at each clinic visit. In our experience, answering the questions by circling the options allows for a visual estimate of symptom burden, with more circles on the “left side” of the page suggesting a low symptom burden and more on the “right side” of the page suggesting a higher symptom burden. When scanned into a patient’s electronic chart, it can be easy to compare the questionnaires from visit to visit so that clinicians can see what is improving or worsening. It is also helpful for patients to see their own prior questionnaire and compare it to the current one so they can see what progress is being made or where we need to keep focusing our attention for treatment, helping them become more active participants in their care and possibly improving their outcomes.

Our pilot program creation and use of the SHFCQ helped to fill a noticeable gap in our clinic assessments. Patient-reported symptoms and general symptom awareness are crucial to adequate evaluation, diagnosis, and management of HF. Jurgens et al. report that “symptoms also drive health care use and are a major contributor to broad patient-reported outcomes such as quality of life” [5]. The World Health Organization defines quality of life as an “individual’s perception of their position in life in the context of the culture and value system in which they live and in relation to their goals, expectations, and standards and concerns.” The term "health-related quality of life" (HRQoL) broadens that definition to include a “functional effect of physical, mental, and social response to the disease and treatment as perceived by the patient” [6]. There is value in allowing patients to self-report their symptoms and rate how those symptoms affect their HRQoL, and we did not feel this was being done well enough in our clinic prior to use of the SHFCQ.

Improving a provider’s symptom assessment can help to improve patient outcomes. One study shows that patients reporting a moderate symptom burden were 82% more likely to have an adverse event within one year, and patients reporting a severe burden of symptoms were more than twice as likely to have an event [7]. Unfortunately, providers are not always good at recognizing or investigating symptoms, and they often perceive them differently from how patients report them [7]. Patient-reported symptom questionnaires have been a common tool used in HF to better aid the clinician in symptom management and determining how those symptoms affect quality of life [8]. These reported symptoms can also help providers determine the patient's NYHA score, which, as mentioned above, can also help with determining prognosis [4]. Our pilot program patients demonstrated that the use of the SHFCQ helped providers delve deeper into specific symptoms and monitor their progress after interventions were done.

Though it has not yet been scientifically validated, our providers found the SHFCQ helpful to improve symptom assessment in our HF clinics. Improved symptom management in HF patients should include a comprehensive assessment of symptoms and knowledge of possible approaches to alleviate the symptoms [7]. The SHFCQ evaluates patient-reported symptom burden and quality of life in a simplified, cost-effective way that could be used easily in clinics to benefit HF patients. Symptom management is a top priority for patients, especially in chronic or end-stage disease. In the Evaluation Study of Congestive Heart Failure and Pulmonary Artery Catheterization Effectiveness (ESCAPE) trial, it was noted that over half of advanced HF patients would trade more survival time for improved symptom control, and almost one quarter of them would “trade nearly all of their remaining time to feel better” [7]. Patient-reported symptoms are one of the best ways to help address the often-distressing symptom burden in HF. Our HF patients have been better able to report their symptoms clearly and concisely and show the burden that those symptoms have on their quality of life. They reported benefits from using the SHFCQ, with many of them sharing their appreciation of feeling that their symptoms were better heard and addressed.

As mentioned, many other patient-reported symptom questionnaires can be used in the HF clinic. The Kansas City Cardiomyopathy Questionnaire (KCCQ) was one of the first questionnaires utilized in HF to better evaluate symptom burden on HRQoL [9-11]. The KCCQ was created by Dr. Pertus in 1996 and was published in 2000 [9]. In 2020, the U.S. Food and Drug Administration formally qualified it as a clinical outcome assessment and recommended it as a primary endpoint in drug trials [12]. In the last 20 years, more questionnaires have also been developed and utilized around the world to monitor patient-reported symptoms, such as the Minnesota Living with Heart Failure (MLHF) [13], Chronic Heart Failure (CHF) [14], Heart Quality of Life (HQoL) [15], and Short-form Health Survey (SfHS) [16]. Despite a variety of tools available for better symptom reporting, there are difficulties with utilizing them in many HF clinics. The KCCQ, MLHF, CHF, and HQoL are licensed questionnaires that can be costly to use in the clinic setting, with costs as high as $15,000 USD annually [9]. The SfHS is 36 questions long and would be more cumbersome to use regularly in the clinic setting, as it would take more time for patients to fill out and for providers to interpret. See Table 4 for a comparative table of the above-mentioned questionnaires. Our providers needed an easy-to-use questionnaire that was free to utilize in our clinics, and we created and piloted the SHFCQ to fill this need.

**Table 4:** Comparison of HF symptom and quality-of-life questionnaires KCCQ: Kansas City Cardiomyopathy Questionnaire [9], MLHFQ: Minnesota Living With Heart Failure Questionnaire [13], CHFQ: Chronic Heart Failure Questionnaire [14], Heart QoL: Heart Quality of Life Questionnaire [15], SF-36: Short-Form Health Survey [16], SHFCQ: Simplified Heart Failure Clinic Questionnaire, HF: heart failure

Instrument	Number of questions	Licensing requirements	Typical cost	Clinical feasibility
KCCQ	23 (12-item short form available)	License required through Outcomes Instruments, Limited Liability Company (LLC)	Variable; project- or patient-based fees	Self-administered, takes 3-5 minutes to complete
MLHFQ	21	University of Minnesota license required	$0-15,000 depending on project type and intended use	Self-administered, takes 5-10 minutes to complete
CHFQ	20	Permission from developers is generally required	Variable	Interviewer-administered, takes 10-20 minutes to complete and additional staff time
HeartQoL	14	Free for most academic and non-commercial use	Usually free for academic use	Self-administered, takes 5 minutes to complete
SF-36	36	RAND version available free; commercial versions require licensing	$0 to several thousand dollars	Self-administered, takes 7-10 minutes to complete, measures general health status rather than HF-specific outcomes
SHFCQ (new)	5	No license needed	Free	Self-administered, takes 3-5 minutes to complete

Although we described only three patient examples of using this questionnaire, we have used it on over 1,000 patients and received positive feedback from many of them; fewer than five patients declined to use it. Our providers elected to continue using it because they found it invaluable in practice. One limitation of our questionnaire is that it’s currently only in paper form and has not yet been digitized. Electronic patient-reported outcome monitoring has been shown to improve physician communication, treatment understanding, and patient satisfaction [17]. Making this questionnaire available within an electronic health system through a digital questionnaire would make it more easily usable by patients and providers. Its other limitation is that it has yet to be validated, which will be the next step for the authors to undertake.

## Conclusions

The pilot patients above, and many more who have used the SHFCQ over the past two years, have shown us that a free, simplified questionnaire is beneficial for managing the care of all types of complex, chronic HF patients in a clinic setting. Providers in our clinics have found it helpful to ease the burden of symptom questioning during the exam, and the SHFCQ has helped focus clinical questioning on the symptoms patients report as most bothersome. Further large-scale validation studies are needed to demonstrate the SHFCQ's validity and reliability compared to other available questionnaires and to make it available freely to other clinicians/clinics. Based on our pilot experience using the SHFCQ in our HF clinics, we believe that using this questionnaire has the potential to help improve patients’ quality of life and decrease untreated symptoms that can lead to emergency department visits, hospitalizations, and increased long-term mortality, similar to other questionnaires, but with no cost to the provider or institution and minimal burden to the patient and provider.

## References

[1] Savarese G, Becher PM, Lund LH, Seferovic P, Rosano GM, Coats AJ (2023). Global burden of heart failure: a comprehensive and updated review of epidemiology. Cardiovasc Res.

[2] Sweitzer NK (2019). Choosing a career in heart failure. Circ Heart Fail.

[3] Savarese G, Lindenfeld J, Stolfo D (2023). Use of patient-reported outcomes in heart failure: from clinical trials to routine practice. Eur J Heart Fail.

[4] Ahmed A (2007). A propensity matched study of New York Heart Association class and natural history end points in heart failure. Am J Cardiol.

[5] Jurgens CY, Lee CS, Aycock DM (2022). State of the science: the relevance of symptoms in cardiovascular disease and research: a scientific statement from the American Heart Association. Circulation.

[6] Gierlaszyńska K, Pudlo R, Jaworska I, Byrczek-Godula K, Gąsior M (2016). Tools for assessing quality of life in cardiology and cardiac surgery. Kardiochir Torakochirurgia Pol.

[7] Alpert CM, Smith MA, Hummel SL, Hummel EK (2017). Symptom burden in heart failure: assessment, impact on outcomes, and management. Heart Fail Rev.

[8] Garin O, Herdman M, Vilagut G (2014). Assessing health-related quality of life in patients with heart failure: a systematic, standardized comparison of available measures. Heart Fail Rev.

[9] (2025). KCCQ: enhancing heart failure care & quality of life. https://www.codetechnology.com/blog/kansas-city-cardiomyopathy-questionnaire-kccq/.

[10] Green CP, Porter CB, Bresnahan DR, Spertus JA (2000). Development and evaluation of the Kansas City Cardiomyopathy Questionnaire: a new health status measure for heart failure. J Am Coll Cardiol.

[11] Spertus JA, Jones PG (2015). Development and validation of a short version of the Kansas City Cardiomyopathy Questionnaire. Circ Cardiovasc Qual Outcomes.

[12] Stogios N, Fezza G, Wong JV, Ross HJ, Farkouh ME, Nolan RP (2021). Current challenges for using the Kansas City Cardiomyopathy Questionnaire to obtain a standardized patient-reported health status outcome. Eur J Heart Fail.

[13] Bilbao A, Escobar A, García-Perez L, Navarro G, Quirós R (2016). The Minnesota living with heart failure questionnaire: comparison of different factor structures. Health Qual Life Outcomes.

[14] Guyatt GH, Feeny DH, Patrick DL (1993). Measuring health-related quality of life. Ann Intern Med.

[15] Oldridge N, Höfer S, McGee H, Conroy R, Doyle F, Saner H (2014). The Heartqol: Part I. Development of a new core health-related quality of life questionnaire for patients with ischemic heart disease. Eur J Prev Cardiol.

[16] Ware JE, Sherbourne CD (1992). The MOS 36-item short-form health survey (SF-36). Med Care.

[17] Yamashita S, Katsumata Y, Kohsaka S (2025). Electronic patient-reported outcome system implementation in outpatient cardiovascular care: a randomized clinical trial. JAMA Netw Open.

